# *Brm* Inhibits the Proliferative Response of Keratinocytes and Corneal Epithelial Cells to Ultraviolet Radiation-Induced Damage

**DOI:** 10.1371/journal.pone.0107931

**Published:** 2014-09-25

**Authors:** Nur Mohammad Monsur Hassan, Nicole Painter, C. Rolfe Howlett, Andrew W. Farrell, Nick Di Girolamo, J. Guy Lyons, Gary M. Halliday

**Affiliations:** 1 Discipline of Dermatology, Bosch Institute, Sydney Medical School, Royal Prince Alfred Hospital and University of Sydney, Sydney, Australia; 2 Department of Pathology and Graduate School of Biomedical Engineering, University of New South Wales, Sydney, Australia; 3 School of Medical Sciences, University of New South Wales, Sydney, Australia; 4 Sydney Head and Neck Cancer Institute, Cancer Services, Royal Prince Alfred Hospital, Sydney, Australia; University of Queensland Diamantina Institute, Australia

## Abstract

Ultraviolet radiation (UV) from sunlight is the primary cause of skin and ocular neoplasia. Brahma (BRM) is part of the SWI/SNF chromatin remodeling complex. It provides energy for rearrangement of chromatin structure. Previously we have found that human skin tumours have a hotspot mutation in *BRM* and that protein levels are substantially reduced. *Brm−/−* mice have enhanced susceptibility to photocarcinogenesis. In these experiments, *Brm−/−* mice, with both or a single *Trp53* allele were exposed to UV for 2 or 25 weeks. In wild type mice the central cornea and stroma became atrophic with increasing time of exposure while the peripheral regions became hyperplastic, presumably as a reparative process. *Brm−/−, Trp53+/−,* and particularly the *Brm−/− Trp53+/−* mice had an exaggerated hyperplastic regeneration response in the corneal epithelium and stroma so that the central epithelial atrophy or stromal loss was reduced. UV induced hyperplasia of the epidermis and corneal epithelium, with an increase in the number of dividing cells as determined by Ki-67 expression. This response was considerably greater in both the *Brm−/− Trp53+/+* and *Brm−/− Trp53+/−* mice indicating that *Brm* protects from UV-induced enhancement of cell division, even with loss of one *Trp53* allele. Cell division was disorganized in *Brm*−/− mice. Rather than being restricted to the basement membrane region, dividing cells were also present in the suprabasal regions of both tissues. *Brm* appears to be a tumour suppressor gene that protects from skin and ocular photocarcinogenesis. These studies indicate that *Brm* protects from UV-induced hyperplastic growth in both cutaneous and corneal keratinocytes, which may contribute to the ability of *Brm* to protect from photocarcinogenesis.

## Introduction

Ultraviolet (UV) radiation from sunlight is the main cause of skin cancer [1], and also causes chronic damage to the eye, including ocular cancer [2]. SWI/SNF is a chromatin-remodeling complex that regulates chromatin structure. It modulates transcription and regulates DNA repair enzyme access to damaged DNA [3]. It is therefore a master regulator of multiple cellular processes and evidence is emerging that several subunits of this complex are tumour suppressor genes [4]. The energy to unravel DNA is supplied by one of two mutually exclusive ATPase subunits of SWI/SNF, *Brm* and *Brg-1* [3]. We have found that human non-melanoma skin cancers (squamous cell carcinomas [SCC] and basal cell carcinomas [BCC]) have a missense hot-spot mutation in the *BRM* gene that is predicted to change amino acid sequence of the protein and inhibit function [5]. In addition, BRM protein was reduced by approximately 10-fold in 100% of the human SCC and BCC that we examined [6]. Functional evidence that *Brm* is a tumour suppressor gene for skin and ocular cancer came from our photocarcinogenesis studies in *Brm*−/− mice [7]. *Brm*−/− mice had enhanced skin and ocular cancer formation compared to wild type (*Brm*+/+) controls when exposed to ultraviolet radiation (UV).

The *TP53* gene is frequently mutated in human skin cancer [8] and is a well-characterized suppressor of UV-induced skin carcinogenesis [9]. As *TP53* mutations occur early during carcinogenesis [10] and loss of a single *Trp53* allele is sufficient to enhance photocarcinogenesis [9] it is possible that any important role for *Brm* as a tumour suppressor gene may occur against a background of at least partial loss of p53 function. Hence we also examined the effect of *Brm* loss on photocarcinogenesis in mice with loss of a *Trp53* allele. Even with this underlying loss of p53 function, *Brm* loss increased the growth rate of early appearing skin cancers [7].

In this study we have examined whether *Brm* loss gives UV irradiated keratinocytes or corneal epithelial cells a growth advantage. We studied mice with both or only a single *Trp53* allele. One of the important molecular mechanisms for protection from UV carcinogenesis is inhibition of UV-induced cell division. This provides cells more time to repair damaged DNA, reducing the incidence of UV mutagenesis, and reduces uncontrolled growth of cells. *Trp53* functions in this process in part by regulating cell growth and apoptosis [11]. Therefore whether *Brm* loss would also affect UV-induced division of cells with only a single *Trp53* allele is of interest. In mice that commenced the irradiation regime with either one or both *Trp53* alleles, *Brm* protected from UV-induced proliferation of both epidermal keratinocytes and corneal epithelial cells.

## Materials and Methods

### Mice

*Brm*−/− and *Trp53*+/− mice were on a C57BL/6 background and were bred and housed at the University of Sydney animal house [7]. This study was carried out in strict accordance with the recommendations in the Australian code of practice for the care and use of animals for scientific purposes by the National Health and Medical Research Council of Australia. The protocol was approved by the Committee on the Ethics of Animal Experiments of the University of Sydney (Permit Number: K14/1-2011/3/5456). All efforts were made to minimize suffering. Mice were supplied with standard chow and water ad libitum. All mice were female and 6 weeks of age at commencement of experiments. Each mouse was ear marked for identification and tested for *Brm* and *Trp53* gene status by PCR in order to establish the genotype of each mouse. Examples and technical details of genotype determination are shown in Figure S1 in File S1. The *Brm−/−* mice used in our studies have been shown to lack functional BRM protein [12]. The *Trp53+/−* mice we used in these studies have been shown to express about half of the protein levels found in wild-type cells [13].

### UV irradiation

A custom built bank of fluorescent tubes consisting of 4 UVA tubes (Philips, CLEO 80w-R, Netherlands) and 2 UVB tubes (Oliphant FL40SE, Oliphant-UV, Adelaide, S.A.) was used for irradiation. Monitoring of spectral intensity was as previously described [7]. Irradiated and un-irradiated groups of mice were shaved weekly on their dorsal trunk. The irradiation source consisted of 0.6% UVC (280–290 nm), 8.6% UVB (290–320 nm) and 90.8% UVA (320–400 nm). The UV dose is reported as the UVB component only but contained the appropriate amount of the other wavebands. An incremental irradiation protocol was used to avoid sunburn while maintaining a damaging dose as the skin adapted to the repeat exposures [14]. Week 1, 250 mJ/cm^2^ Monday and Friday; week 2, 250 mJ/cm^2^ Monday, Wednesday and Friday; week 3, 300 mJ/cm^2^ Monday, Tuesday, Thursday and Friday; week 4, 400 mJ/cm^2^ on the same days, weeks 5–25, 500 mJ/cm^2^ on the same days.

### Tissue sampling, preparation and measurement of epidermal or corneal epithelium thickness

Eyes and irradiated dorsal trunk skin were removed from euthanized mice 24 h after the final irradiation. Where possible both eyes were removed but in some cases one eye was damaged during removal and was excluded from the study. The tissues were fixed in 10% neutral formalin (skin for 24 h, eyes for 48 h) before transfer into 70% ethanol, and left for at least 24 h before processing. Tissues were embedded in paraffin and sections (skin 5 µm, eye 4 µm) cut onto coated glass slides (Superfrost Plus, Lomb Scientific Pty Ltd. Sydney, Australia). Slides were dried overnight at 45°C. Hematoxylin and eosin stained sections (Surgipath Leica Biosystems, Richmond IL, USA) were scanned using the Aperio Scanscope XT (Aperio Technologies, Sandigo, USA). ImagePro viewing software was used for the measurement of epidermal and corneal epithelial thickness in 10 and 5 (respectively) randomly selected 200x magnification fields. Counts in the corneal epithelium were performed between limbus to limbus in transverse tissue sections. The observer was masked during analysis.

### Immunohistochemistry

Sections from the paraffin blocks were incubated at 60°C for 30 min, then de-waxed by immersion in xylene, then through a series of ethanol baths, 100%, 95% and 70%. Antigen retrieval was performed by microwaving tissues in citrate buffer (pH 6) for 12 min. Endogenous peroxidase was blocked by incubating sections in 0.3% H_2_O_2_ in methanol for 10 min at room temperature and this was followed by blocking for non-specific protein binding by incubation for 20 min in 10% normal goat serum in tris-buffered saline (TBS). All antibodies were diluted in TBS. Rabbit polyclonal antibody to murine Ki-67 (15580, Abcam, Cambridge, MA, USA used at 1∶1000 dilution) was added to the sections and incubated at room temperature for 60 min followed by rinsing in TBS-Tween 20 (0.05% Tween 20; Sigma, St Louis, MO, USA) wash buffer thrice over 2 min. Biotinylated goat anti-rabbit IgG (Vectastain Elite ABC kit [rabbit IgG] in TBS was added for 30 min at room temperature followed by rinsing thrice over 2 min with TBS-Tween 20. The chromagen (Impact Nova red solution, Vector Labotories Cat no SK-4805, Burlingame, CA, USA) was then added for 11 min to visualize the immunoreactivity. The slides were then rinsed in water for 2–5 min, dehydrated through ethanol to 100% ethanol, and cleared in xylene × 2 for 5 min. Specificity controls included omitting the primary antibody and a control rabbit IgG (Dako, Glostrup, Denmark). Digital images were taken of the stained sections using an Olympus BX51 microscope (Olympus, Melbourne, Australia). The number of positive cells was counted from digital images and the length of each section determined using Image J64 (Wayne Rasband, National Institutes of Health, USA) for expression of data as positive cells per mm tissue length. For skin, at least 15 digital photomicrographic images for each mouse were assessed and a mean of these used as the value for that mouse. For the eyes, at least 5 random images per eye were assessed. The images were analysed in a blinded manner.

### Data Analysis

Data was analysed using one-way ANOVA with Holm-Sidak’s multiple comparison test (Prism 6; GraphPad Software Inc., San Diego, CA, USA). A P value of <0.05 was regarded as significant and data for individual mice is shown in the figures. Data descriptions in the text are means and SEM or differences in means between data sets.

## Results

### *Brm* protects epidermal keratinocytes from UV-induced proliferation

#### Epidermal thickness and Ki-67+ cells in mice with both *Trp53* alleles

**Figure 1:**
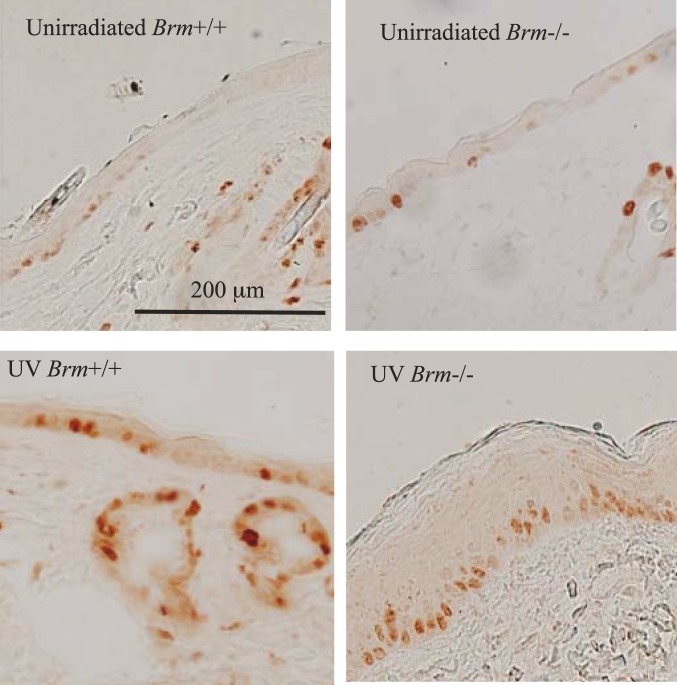
Representative photomicrographs of skin sections stained for Ki-67 in mice with both Trp53 alleles (Trp53+/+) that had been irradiated for 25 weeks. Ki-67+ cells can be seen as brown/red stained cells.

**Figure 2:**
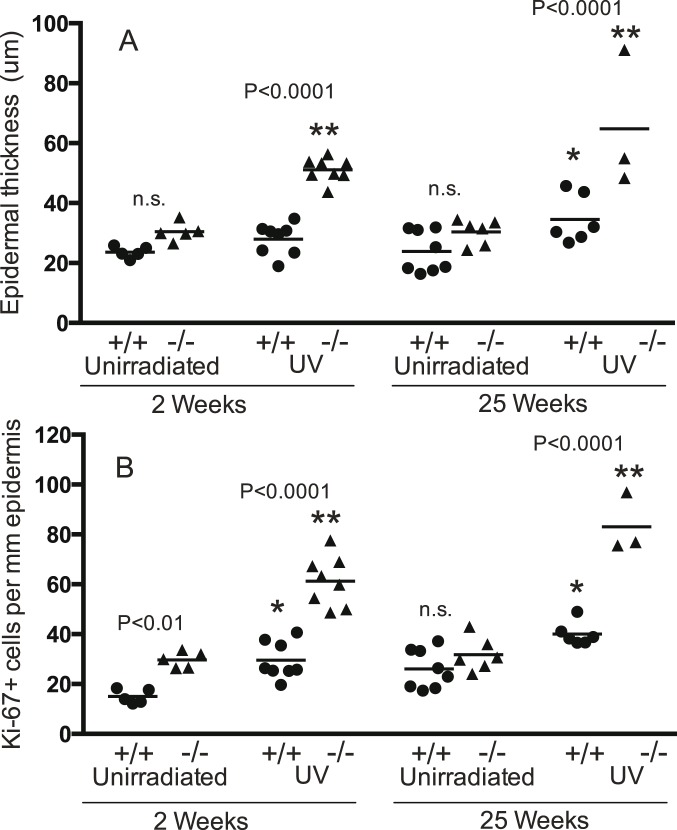
*Brm* protects keratinocytes from UV-induced proliferation. Groups of *Brm* wildtype (+/+, closed circles) or knockout (−/−, closed triangles) mice on a *Trp53* wildtype background were exposed to UV radiation for 2 or 25 weeks. Unirradiated controls were age matched to the 2 or 25 week irradiated groups. Back skin was removed from the UV irradiated region of all groups and (A) epidermal thickness and (B) Ki-67+ keratinocytes were determined. The numerical P values centred between the wildtype and knockout groups indicate statistical comparison between these genotypes for each treatment (n.s. -not significant; ANOVA). Significance of UV effect compared to relevant unirradiated group shown as *(P<0.05) or **(P<0.001). Lack of indication of statistical difference indicates not significant (ANOVA). Mean shown as horizontal line.

*Brm*−/− and wild type mice with both *Trp53* alleles intact were irradiated for 2 or 25 weeks and then examined for epidermal thickness and Ki-67+ within the epidermis (Fig. 1, 2). Unirradiated controls were age matched to the 2 and 25-week groups and treated identically, including shaving, except for irradiation. In the absence of UV there were no significant differences in epidermal thickness between *Brm*−/− and wild type mice at either time. Two weeks of irradiation was not sufficient to cause epidermal hyperplasia in the wild type mice but significantly increased epidermal thickness in the *Brm*−/− mice from a mean (SEM) of 30.44 (1.39) to 51.15 (1.37) µm. The difference between the 2 week irradiated *Brm*−/− and wild type mice was statistically significant. By 25 weeks of UV, epidermal hyperplasia became evident in the wild type mice with a significant increase from 23.85 (2.44) to 34.55 (3.29). Even with this extended UV irradiation, the response in the *Brm*−/− mice was significantly greater with a doubling in epidermal thickness from 30.35 (1.72) to 64.79 (13.29). Therefore the *Brm*−/− mice were more sensitive to UV-induced epidermal hyperplasia than controls (Fig. 2A).

Ki-67 expression confirmed that UV increased the frequency of cell divisions in *Brm*−/− murine epidermis to a greater extent than in controls (Fig. 2B). Unirradiated *Brm*−/− epidermis had significantly greater numbers of dividing cells at the 2-week time point (increase of 14.7 cells/mm epidermis), but the genotypes were not different by 25 weeks. After 2 weeks of irradiation, the number of Ki-67+ cells was significantly increased in both wild type (increase of 14.5 cells/mm) and *Brm*−/− (increase of 31.5) mice. The response in the *Brm*−/− mice was statistically significantly greater than in the controls. This difference was maintained at 25 weeks of irradiation with a significant increase in both mouse genotypes but with the response in the *Brm*−/− mice (increase of 51.3 cells/mm) being significantly greater than the controls (increase of 14.0).

#### Epidermal thickness and Ki-67+ cells in mice with loss of a single *Trp53* allele

**Figure 3:**
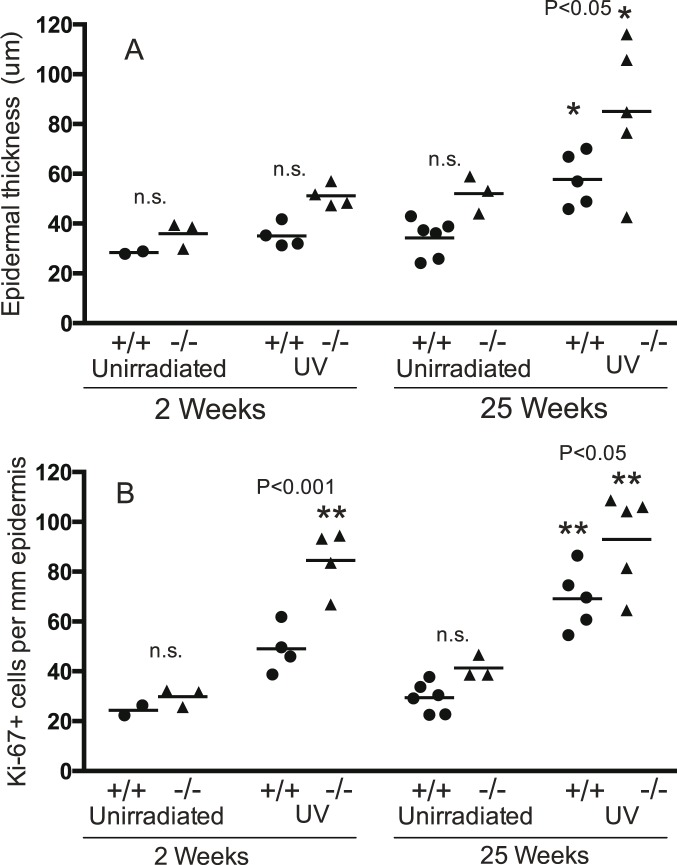
*Brm* protects keratinocytes with only a single *Trp53* allele from UV-induced proliferation. Legend the same as for Fig. 2 except that all mice had only a single *Trp53* allele (*Trp53*+/−).

The function of p53 is frequently lost in human skin tumours, and we have previously determined the effect of *Brm* loss on photocarcinogenesis in mice with loss of a single *Trp53* allele. Therefore these experiments included mice with a single *Trp53* allele (*Trp53*+/−, Fig. 3A). There were no significant effects of *Brm* loss on epidermal thickness in un-irradiated mice at either time. Two weeks of UV did not induce significant epidermal hyperplasia in either *Brm*+/+ *Trp53*+/− or *Brm*−/− *Trp53*+/− mice. However 25 weeks of UV caused a significant increase in both strains. The epidermal thickness after 25 weeks of UV in the *Brm*−/− *Trp53*+/− mice (85.08±12.79 µm) was significantly greater than the *Brm*+/+ *Trp53*+/− mice (57.70±4.76 µm).

Ki-67 expression in the epidermis of unirradiated *Brm*−/− *Trp53*+/− mice was not significantly different to the *Brm*+/+ *Trp53*+/− group at either the 2 or 25-week time points. UV for 2 weeks significantly increased the number of Ki-67+ cells in *Brm*−/− *Trp53*+/− mice (increase of 54.7 cells/mm) but not in the *Brm*+/+ *Trp53*+/− mice and there was a significant difference between the genotypes. By 25 weeks of irradiation both genotypes developed significant increases, but the number of Ki-67+ cells was significantly higher in *Brm*−/− *Trp53*+/− (increase of 51.6 cells/mm) compared to *Brm*+/+ *Trp53*+/− mice (increase of 39.8) (Fig. 3B). Therefore even with loss of a single *Trp53* allele, additional loss of *Brm* increased the number of keratinocytes that proliferated in response to UV.

#### Supra-basal dividing cells in epidermis

**Figure 4:**
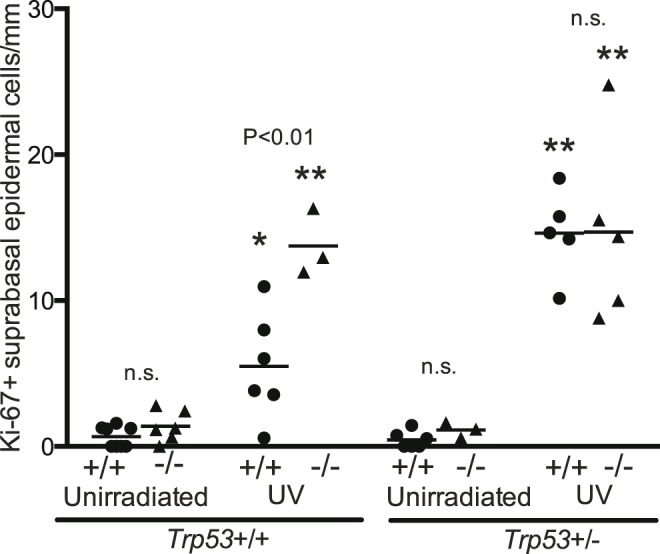
*Brm* protects suprabasal epidermal cells from UV-induced proliferation. Groups of *Brm* wildtype (+/+, closed circles) or knockout (−/−, closed triangles) mice that had either both (*Trp53*+/+) or a single (*Trp53*+/−) *Trp53* allele were exposed to UV radiation for 25 weeks. Unirradiated controls were age matched. Skin sections were stained for Ki-67+ cells. The number of Ki-67+ cells that were two or more cells above the base of the epidermis were determined. Each point represents a single mouse. Mean shown as horizontal line. Statistical comparisons as described in Fig. 2.

In the 25 week irradiated groups there was a large number of Ki-67+ cells more than 2 cells above the epidermal base. All 4 irradiated genotypes had significantly higher numbers of supra-basal Ki-67+ cells than the corresponding unirradiated genotype (Fig. 4). The irradiated *Brm*−/− *Trp53*+/+ group was significantly higher than the *Brm*+/+ *Trp53*+/+ group. This indicates that *Brm* loss enabled cell division to occur in more differentiated cells further from the basement membrane.

### *Brm* protects corneal epithelial cells from UV-induced proliferation

We also examined the cornea as this is the other external surface directly exposed to UV. In addition to the 2 and 25 week irradiation groups that were analysed, a small number of wild type (*Brm*+/+ *Trp53*+/+) mice were irradiated for different times enabling the development of eye pathology to be documented. A detailed description can be found in the supporting information information (File S1) and is summarized here. No histopathological changes to the cornea were observed after 2 weeks of UV. At 5 weeks the epithelium displayed minor changes centrally that led to central atrophy and parakeratosis. Eventually central ulceration occurred in a third of the eyes examined while peripheral regions of corneal epithelium were hyperplastic often containing metaplastic foci.

Histological changes resulting from 25 weeks of UV exposure were compared between the genotypes (Table 1). Loss of *Brm* (*Brm*−/− *Trp53*+/+) increased the number of eyes showing no discernable damage with a corresponding decrease in the number of eyes with atrophy or regions of ulceration. Hyperplasia and dysplasia were increased. Loss of *Brm* increased regeneration or corneal epithelial division in response to UV. Damage was obvious in all eyes of mice with loss of a single *Trp53* allele (*Brm*+/+ *Trp53*+/−), but with higher levels of metaplasia, hyperplasia and dysplasia compared to the wild type mice. There was decreased atrophy but a similar level of ulceration to the wild type mice occurred. Hence, similarly to *Brm*−/−, the *Trp53*+/− mice had increased regeneration of epithelial cells so that atrophy was present in less of the eyes examined. The *Brm*−/− *Trp53*+/− group contained greater numbers of eyes with hyperplasia or dysplasia but displayed similar levels of atrophy to the *Brm*−/− or *Trp53*+/− mice. The pattern of ocular change was consistent with *Brm* loss on either a *Trp53*+/+ or *Trp53*+/− background increasing UV-induced proliferation. Consequently these findings were investigated in more detail.

**Table 1:** Histopathological assessment of eyes in mice irradiated with UV for 25 weeks.

	*Trp53*+/+		*Trp53*+/−	
	*Brm*+/+	*Brm*−/−	*Brm*+/+	*Brm*−/−
**Corneal Epithelium Central**				
No change	0%	25%	0%	0%
Atrophy	80%	38%	43%	38%
Ulcer	33%	0%	29%	25%
Metaplasia	40%	25%	71%	50%
Hyperplasia	20%	38%	50%	63%
Dysplasia	0%	13%	7%	25%
**Corneal Stroma Central**				
Resident Stroma: 25% or more	13%	13%	14%	63%
Resident Stroma: Minimum	53%	88%	86%	38%
Resident Stroma: None (complete necrosis)	33%	0%	0%	0%
Vascular proliferation beyond expected reparative FV^1^	0%	0%	0%	50%
**Reparative Fibrovascular tissue (FV)** 2				
FV in peripheral zones	60%	75%	21%	25%
FV entered &/or breached central zone	40%	25%	79%	75%
Excessive with large luminal vessels^3^	0%	13%	57%	50%
Excessive vascular proliferation^3^	0%	13%	50%	50%

Number of eyes examined - 15 for *Brm*+/+ *Trp53*+/+; 8 for *Brm*−/− *Trp53*+/+; 14 for *Brm*+/+ *Trp53*+/−; 8 for *Brm*−/− *Trp53*+/−.

FV – Fibrovascular.

1 Megalocytic cells were observed in these corneas.

2 Usually accompanied by some inflammatory cells.

3 A few megalocytic cells also present.

### Corneal thickness and Ki-67+ cells in mice with both *Trp53* alleles

**Figure 5:**
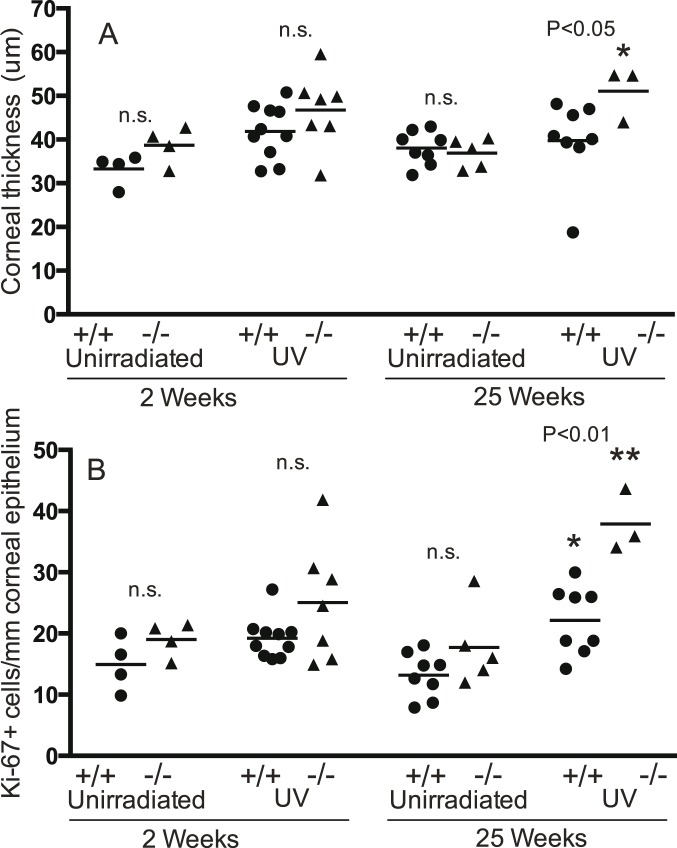
*Brm* protects corneal epithelial cells from UV-induced proliferation. Groups of *Brm* wildtype (+/+, closed circles) or knockout (−/−, closed triangles) mice on a *Trp53* wildtype background were exposed to UV radiation for 2 or 25 weeks. Unirradiated controls were age matched to the 2 or 25 week irradiated groups. Eyes were removed and (A) corneal epithelial thickness, (B) Ki-67+ cells in the corneal epithelium were determined. Each point represents a single mouse. Where both eyes were available for a mouse the mean of the two eyes is used. The numerical P values centred between wildtype and knockout groups indicate statistical comparison between these genotypes for each treatment (n.s. - not significant; ANOVA). Significance of UV effect compared to relevant unirradiated group shown as *(P<0.05) or **(P<0.001). Lack of indication of statistical difference indicates not significant (ANOVA). Mean shown as horizontal line.

There were no significant differences in corneal thickness between the unirradiated genotypes at either time point, or after 2 weeks exposure (Fig. 5A). However after 25 weeks of UV there was a significantly increased corneal thickness in *Brm*−/− (increase of 14.2 µm above unirradiated group) but not *Brm*+/+ mice (increase of 1.7). This statistically significant difference (Fig. 5A) between the irradiated genotypes was consistent with the histopathological observations of reduced atrophy and increased hyperplasia in the *Brm*−/− mice.

**Figure 6:**
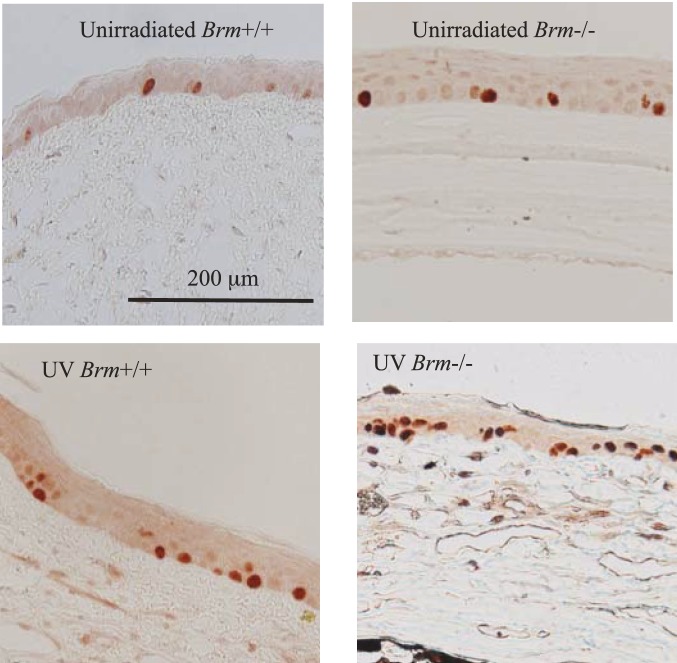
Representative photomicrographs of corneal sections stained for Ki-67 in mice with both Trp53 alleles (Trp53+/+) that had been irradiated for 25 weeks. Ki-67 cells can be seen as brown/red stained cells.

Ki-67+ cell numbers confirmed that *Brm* regulates corneal epithelial cell division induced by UV (Fig. 5B, 6). There were no differences between the un-irradiated genotypes at either time point. Similarly to corneal thickness, Ki-67+ cell numbers were not altered in either genotype by exposure to 2 weeks of UV. Irradiation for 25 weeks significantly increased the number of Ki-67+ cells in both genotypes with the response in *Brm*−/− mice (increase of 20.2 cells/mm above the un-irradiated group) significantly greater than in *Brm*+/+ mice (increase of 9.0). This is consistent with the histopathology assessment (Table 1) that loss of *Brm* increased proliferation/regeneration of epithelial cells after 25 weeks of UV.

### Corneal thickness and Ki-67+ cells in mice with loss of a single *Trp53* allele

**Figure 7:**
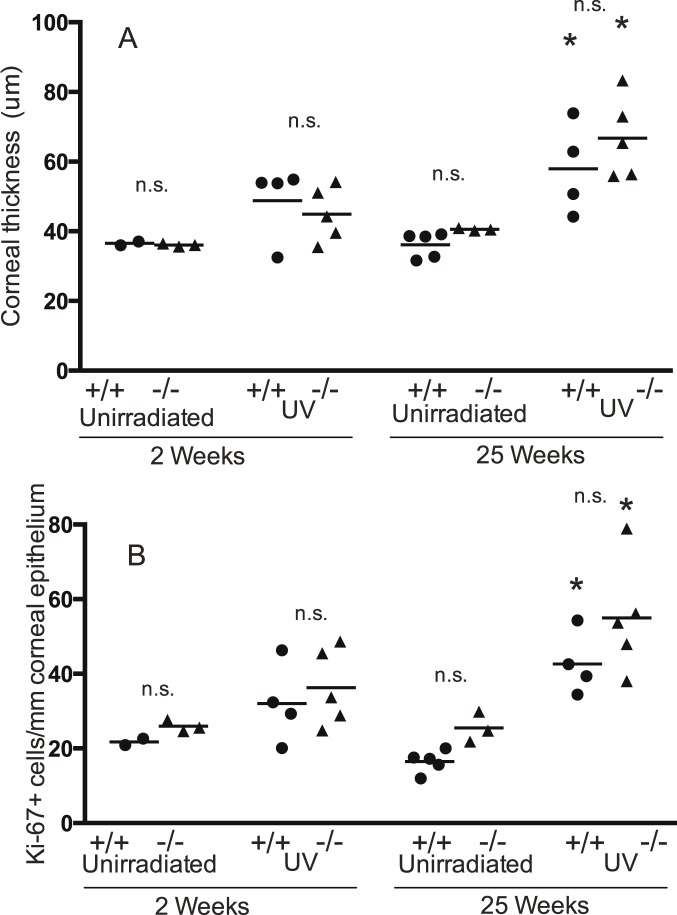
UV-induced proliferation of corneal epithelial cells with only a single *Trp53* allele. Legend the same as for Fig. 5 except that all mice had only a single *Trp53* allele (*Trp53*+/−).

There were no significant differences between genotypes for any un-irradiated or UV irradiated groups for either corneal thickness (Fig. 7A) or Ki-67+ cells (Fig. 7B) in mice with loss of a single *Trp53* allele. UV exposure for 25 but not 2 weeks caused a significant increase in both parameters for both genotypes (increased corneal thickness of 21.8 µm for *Brm*+/+ *Trp53*+/− and 26.22 µm for *Brm*−/− *Trp53*+/− mice, increased Ki-67+ cells/mm of 26.2 for the *Brm*+/+ *Trp53*+/− and 29.5 for the *Brm*−/− *Trp53*+/− mice).

### Supra-basal dividing cells in corneal epithelium

**Figure 8:**
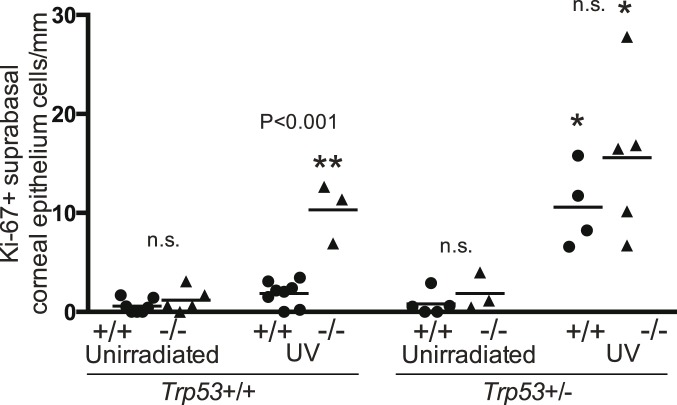
*Brm* protects suprabasal corneal epithelial cells from UV-induced proliferation. Groups of *Brm* wildtype (+/+, closed circles) or knockout (−/−, closed triangles) mice that had either both (*Trp53*+/+) or a single (*Trp53*+/−) *Trp53* allele were exposed to UV radiation for 25 weeks. Unirradiated controls were age matched. Eyes were removed and sections stained for Ki-67+ cells. The number of Ki-67+ cells that were two or more cells above the base of the corneal epithelium were determined. Each point represents a single mouse with the mean shown as a horizontal line. Statistical comparisons as described for Fig. 2.

Many of the Ki-67+ cells in the 25 week irradiated groups were more than 2 cells above the base of the corneal epithelium. Therefore these supra-basal Ki-67+ cells were counted (Fig. 8). For mice with both *Trp53* alleles intact, the UV irradiated *Brm*−/− but not the *Brm*+/+ group was significantly higher than the corresponding un-irradiated genotype (Fig. 8). Loss of a single *Trp53* allele resulted in both the *Brm*+/+ *Trp53*+/− and the *Brm*−/− *Trp53*+/− irradiated groups being significantly higher than the un-irradiated controls. This indicates that *Brm* loss enabled cell division to occur in a more disorganized manner higher in the corneal epithelium.

### *Brm* protects corneal stromal cells from UV-induced proliferation

Analysis of the skin sections for UV-induced histopathological changes in the dermis did not reveal any obvious differences in cell content or structure between the genotypes. The expected changes in response to UV were not noticeably different between the genotypes.

**Figure 9:**
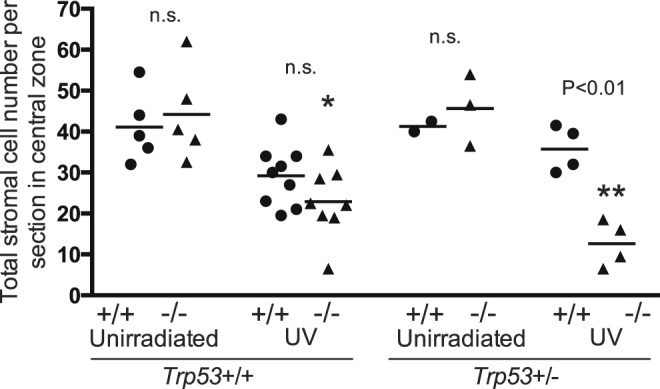
*Brm* protects from UV-induced depletion of corneal stromal cells. Groups of *Brm* wild type (+/+, closed circles) or knockout (−/−, closed triangles) mice that had either both (*Trp53*+/+) or a single (*Trp53*+/−) *Trp53* allele were exposed to UV radiation for 2 weeks. Unirradiated controls were age matched. Eyes were removed and sections stained with H&E. The number of corneal stromal cells in the central region between the iris up to a distance of 0.5 mm were counted. Each point represents a single mouse with the mean shown as a horizontal line. Statistical comparisons as described for Fig. 2.

Histopathological examination of corneal stroma in the 2 week UV group showed scattered loss of cells but no other noticeable damage. There were no significant differences between the unirradiated *Brm*+/+ *Trp53*+/+ and *Brm*−/− *Trp53*+/+ groups, or between the unirradiated *Brm*+/+ *Trp53*+/− and *Brm*−/− *Trp53*+/− groups (Fig. 9). UV statistically significantly reduced the number of stromal cells in *Brm*−/− (UV-induced reduction of 21.3 cells) but not *Brm*+/+ mice (non-significant decrease of 11.9 cells) with both *Trp53* alleles intact although there was no significant difference between these genotypes. The irradiated *Brm*−/− *Trp53*+/− group had significantly fewer stromal cells (significant UV-induced reduction of 33.0 cells) than the *Brm*++ *Trp53*+/− mice (non-significant decrease of 5.5 cells). This therefore suggested that in the stroma, *Brm* protected from 2 weeks of UV-induced damage. Eye damage was noticed in the corneal stroma at an earlier time than in the corneal epithelium.

**Figure 10:**
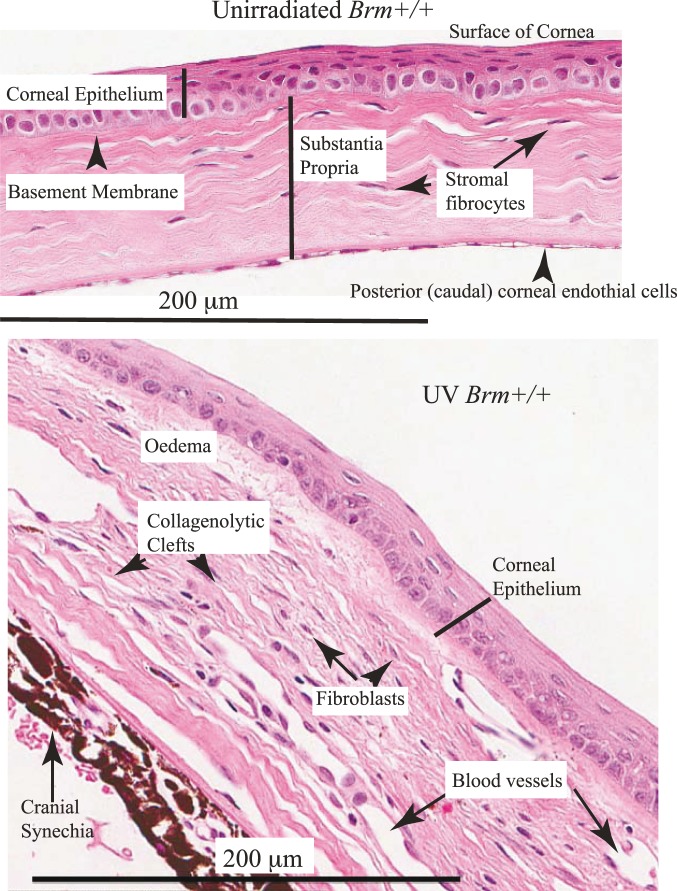
Photomicrographs representing the histological features of the eyes from 25 week unirradiated and UV irradiated Trp53+/+Brm+/+ mice. Sections stained with H&E. Cranial synechia containing proliferating iris fibrovascular tissue, with melanin containing cells. The UV irradiated tissue had odema which was marked particularly under the epithelium.

The time course for histopathologically observed damage to the corneal stroma in wildtype mice is described in supporting information (File S1). In summary, initially there was loss of cells and oedema. With time this resulted into centrally positioned collagenolytic clefts and voids. Simultaneously fibrovascular proliferative twigs formed and extended presumably by migration towards the centrally damaged zones. The proliferative fibrovascular tissue often had a sprinkling of inflammatory reactive cells (Fig. 10).

**Figure 11:**
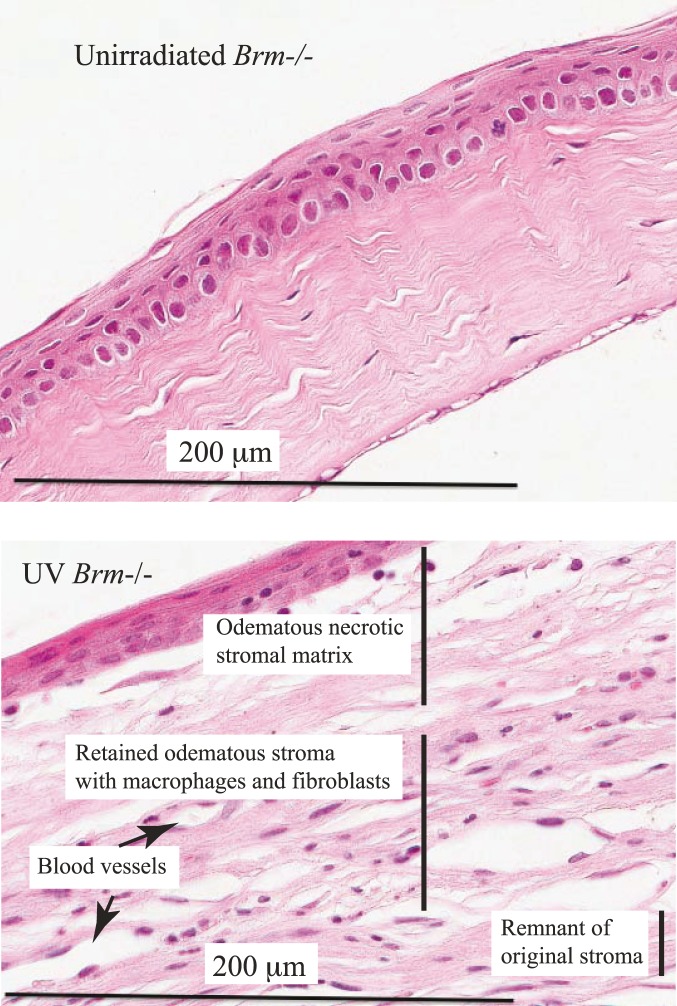
Photomicrographs representing the histological features of the eyes from 25 week unirradiated and UV irradiated Trp53+/+Brm−/− mice. Sections stained with H&E. The UV irradiated tissue had odema which was marked particularly under the epithelium. The unirradiated photomicrograph does not have additional labels as they would be similar to the unirradiated Trp53+/+Brm+/+ figure.

Stroma in the different genotypes exposed to UV for 25 weeks were compared (Fig. 11; Table 1) and appeared to show a reparative program to match that of corneal epithelium. While the UV exposed *Brm*−/− mice contained fewer stromal cells at 2 weeks as described above, both *Brm*−/− and *Trp53*+/− mice had an increased regenerative response by 25 weeks. While 33% of wild type mice had complete necrosis of the stroma by 25 weeks, all mice from the other 3 genotypes had at least minimal stroma retained and more excessive vascular proliferation. Furthermore, 50% of eyes from the *Brm*−/− *Trp53*+/− group had greater than expected vascular proliferation.

The stroma of *Brm*−/− *Trp53*+/− mice was the least damaged of the other genotypes. The original caudal stroma remained present and this genotype contained the largest number of eyes with more than 25% of the stroma remaining. Reparative vascular proliferation was excessive (beyond the wild type) in 50% of the *Brm*−/− *Trp53*+/− mice, which was greater than any other genotype. In one eye there was a more aberrant vascular formation nearby to which were a few megalocytic cells. In one eye the neo-angiogenesis was most proliferative. Megalocytes were sprinkled in these eyes and also observed in the eye with a haemangioma.

In summary, the pattern in the stroma was that both *Brm*−/− and *Trp53*+/− mice, and particularly the combined knockout had increased reparative processes, which included the fibrovascular response resulting in reduced loss of stromal cells. This is consistent with both of these genes being protective from proliferative responses that occur as a result of UV radiation. Thus in the absence of either *Brm* or *Trp53*, an increased proliferation/reparative process lead to lower levels of central necrosis and increased fibrovascular responses.

## Discussion

Our recent studies indicate that *Brm* is a tumour suppressor gene that protects from UV-induced skin and ocular carcinogenesis. *Brm* knockout mice have increased sensitivity to both skin and ocular photocarcinogenesis [7]. Unexpectedly the *Brm*−/− mice were resistant to UV-induced immunosuppression, which would be expected to protect from photocarcinogenesis indicating that different mechanisms are involved. In human skin cancers, the *BRM* gene contains a hotspot mutation that was predicted to affect function [5], and protein expression is decreased compared to normal skin [6]. In the present study we show that *Brm* protects from UV-induced proliferation of keratinocytes and ocular epithelial cells even when one *Trp53* allele is knocked out. Additionally *Brm* reduces the proliferative response of stromal cells in the UV-irradiated eye. The greater reparative response in the stroma of UV-irradiated *Brm*−/− mice may contribute to the higher proliferation of epithelial cells and provide greater support to development of ocular neoplasia. These events may partially explain the functional role of *Brm* in protecting from skin and ocular cancer.

The Ki-67 antigen, a high molecular weight non-histone protein, is generally accepted to be a reliable marker of many types of proliferating cells [15] [16]. It is a nuclear structure expressed during all phases of the cell cycle (G_1_, S, G_2_ and mitosis) but not by cells that are failing to undergo division. While Ki-67 redistributes throughout the nucleus and cytoplasm during cell division, it appears to be required for progression through the cell cycle [17] [16] [18]. The number of Ki-67 expressing keratinocytes and corneal epithelial cells was higher in irradiated *Brm* −/− mice than in irradiated *Brm*+/+ controls. This was observed with as little as 2 weeks of UV in keratinocytes. UV-induced cell proliferation remained elevated after 25 weeks of UV in both the skin and eye, which is at about the time that skin and ocular tumours commenced to appear in our photocarcinogenesis study using mice of these genotypes [7]. Furthermore, in both keratinocytes and corneal epithelial cells, UV-induced proliferation was more disorganized in the *Brm*−/− mice, with substantial numbers of proliferating cells in the suprabasal region. In unirradiated, and even UV irradiated wild type mice, proliferation was largely restricted to basal cells. Examination of epidermal and corneal thickness gave consistent data to assessment of Ki-67+ cells showing that *Brm* protects from UV-induced hyperplasia of the respective tissues. Therefore *Brm* protects from UV-induced proliferation during the entire time course of photocarcinogenesis without any indication of adaptive mechanisms capable or compensating for loss of *Brm*.

Similar findings were observed in mice with both wild type *Trp53* alleles intact and also in mice with loss of a single *Trp53* allele. This suggests that even with partial loss of p53 function, *Brm* protects from UV-induced cellular proliferation.

Thickening of the corneal stroma was also evident in UV irradiated *Brm*−/− mice. Initially, after 2 weeks of UV, there was increased UV-induced damage in the *Brm*−/− mice as indicated by the more severe loss of central corneal stromal cells. With increasing time, the central corneal stromal cells were also lost from *Brm*+/+ mice as previously reported for 129 mice [19], and the damage to this region of the eye became more pronounced with increasing time of irradiation. Either this loss, or chronic UV for 25 weeks then caused an enhanced reparative or proliferative fibrovascular response arising from peripheral zones. The *Brm*−/− mice, with either both or a single *Trp53* allele had an even greater peripheral reparative response resulting in protection of the central resident stroma. Thus *Brm* also appears to protect the stroma from UV-induced hyperproliferation or excessive regeneration. The enhanced proliferative response in UV-irradiated stroma of the eye may contribute to the enhanced ocular photocarcinogenesis in these mice. This stromal response may even contribute to the hyperproliferation observed in corneal epithelial cells by the production of growth factors or by some other cell to cell mechanism.

*Brm* loss has also been shown to enable hyperplasia in prostate cells, which was associated with the cell cycle regulator E2F1 [20]. The liver cells of Brm−/− mice proliferate at an enhanced rate indicative that this gene regulates hepatic cellular proliferation [12]. Furthermore, primary mouse embryonic fibroblasts (MEFs) cultured from *Brm*−/− mice proliferated faster than those from wild-type mice. In our experiments there was a small increase in Ki-67+ epidermal cells in unirradiated *Brm*−/− *Trp53*+/+ mice at 2 weeks. However this did not result in observable thickening of the epidermis. Moreover, this was not evident in older mice, or in mice with loss of a single *Trp53* allele or in corneal epithelial cells. Hence in the absence of *Brm*, proliferation of keratinocytes and corneal epithelia cells appears to be well controlled in the absence of an external stimulus such as UV radiation. Redundancies in cell cycle machinery have been reported to enable *Brm*−/− murine fibroblasts to maintain relatively normal control of the cell cycle under normal growth conditions. However the steps are less tightly regulated so that the stress of serum starvation alters re-entry into the cell cycle upon serum stimulation [21].

Following irradiation with 1 mJ/cm^2^ UV from a Stratalinker 2400 (Stratagene), *Brm*−/− MEFs proliferated at a faster rate than MEFs from wild type mice [12]. In these experiments the UV exposure substantially decreased proliferation compared to un-irradiated cells, with the inhibitory effect less substantial in the *Brm*−/− MEFs. This differs from our data, which found UV to enhance, rather than inhibit proliferation compared to un-irradiated cells. There could be many reasons for this discrepancy. We performed chronic irradiation of whole mice as opposed to a single UV exposure to cultured cells. We examined different cell types that are on the surface of the body and therefore usually exposed to UV from sunlight. We also used a different UV irradiation source. The Stratalinker was used to induce DNA damage as it is used for UV crosslinking of DNA and RNA. It has a maximal output at 254 nm, which is within the UVC range. Our goal was quite different, being to simulate solar UV radiation to study chronic effects that lead to skin and ocular carcinogenesis. Our UV source emitted 90% UVA with a peak irradiance at 360 nm and did not emit any wavelengths as low as 254 nm. Consequently, the biological effects of these two UV sources are very different, and up to 500 fold different UV doses were used in our studies. Despite these differences our data is fundamentally consistent with this previous report that *Brm* regulates cell proliferation and that *Brm* knockout gives cells a proliferative advantage following exposure to UV. However our studies contrast with the finding of decreased survival resulting from knockout of the *Brm* analogue *psa-4* in the nematode Caenorhabditis elegans (C. elegans) exposed to UVB [22]. The reason for this is not obvious, but it may be due to differences in sensitivity to UVB between C. elegans and mammalian keratinocytes. C. elegans were killed by the very low dose of 12–16 mJ/cm^2^ UVB, while even doses of 4,000 mJ/cm^2^ UVB have little effect on viability of human keratinocytes [23].

Cell division prior to repair of DNA damage can lead to a mutation. Therefore the increased cell division seen in these studies in *Brm*−/− mice would reduce the time available for repair of UV damaged DNA. Additionally, cell division in the UV-irradiated *Brm*−/− mice occurred at higher levels in both the epidermis and corneal epithelium. These more superficial suprabasal positioned cells would be exposed to higher amounts of UV than the more protected cells at the basement membrane and therefore could be particularly susceptible to UV-induced mutagenesis. These events could contribute to the augmented photocarcinogenesis observed in our previous study [24]. Chromatin remodeling by SWI/SNF is important for efficient repair of damaged DNA [3]. Downregulation of *BRM* has been shown to decrease repair of DNA damaged by the genotoxic agent cisplatin in human cancer cells [25]. Additionally, *Brm*−/− mice responding to serum stimulation following serum starvation display genetic instability with frequent loss of chromosome stability [21]. It is not known whether loss of *Brm* also inhibits repair of UV-induced genetic damage. However the combination of DNA repair defects and UV-induced hyperproliferation of keratinocytes and corneal epithelia cells would be expected to enable an increased frequency of UV-induced DNA damage becoming fixed into mutations. These molecular events are likely to be responsible for the enhanced photocarcinogenesis that we have observed in *Brm*−/− mice. These events would also give a growth advantage to those tumour cells that have lost *Brm* function so that they would outgrow tumour cells with normal *Brm* function.

Previously we have found that *Brm* protects from UV-induced skin and ocular carcinogenesis. To make these experiments relevant to human skin and ocular cancer, we used a UV spectrum that simulated sunlight and chronic exposure with doses that did not cause excessive sunburn damage. This therefore mimics the exposure of humans to sunlight during their normal daily activities. Our goal was to examine the molecular events by which *Brm* protects from photocarcinogenesis. We had previously shown that *Brm−/−* does not affect UV induction of apoptotic sunburn cells [7] and therefore examined markers of cell proliferation. The absence of *Brm* increased UV-induced keratinocyte and corneal epithelial cell proliferation. This occurred in mice that started the experiments with both or only a single *Trp53* allele in order to mimic the loss of this important tumour suppressor gene in human UV carcinogenesis. *Brm* protection from UV-induced cellular hyperproliferation could contribute to its ability to protect from photocarcinogenesis.

## Supporting Information

File S1 **Genotyping of individual mice.** Examples of PCR gels with the expected PCR products obtained during genotyping of individual mice. Each mouse was genotyped. The allele being detected is shown as heading to each panel with the expected band size. The genotype of the mouse is shown below each lane. HyperLadder II DNA Ladder (Bioline (Aust) Pty Ltd, Sydney, Australia) was used (M). Genomic DNA isolated from mouse tails PCR-amplified with specific primers. The PCR products were resolved on 1.5% AMRESCO agarose (Astral Scientific Pty Ltd, Sydney, Australia) and then visualized by SYBR Safe DNA Gel (Invitrogen, Carlsbad, CA, USA) staining.
(DOCX)
