# Supplementary material for: Brm Inhibits the Proliferative Response of Keratinocytes and Corneal Epithelial Cells to Ultraviolet Radiation-Induced Damage
Source: PLoS One. 2014 Sep 25;9(9):e107931. doi: 10.1371/journal.pone.0107931 (PMC4177874; doi:10.1371/journal.pone.0107931)
Supplement: File S1 — Genotyping of individual mice. Examples of PCR gels with the expected PCR products obtained during genotyping of individual mice. Each mouse was genotyped. The allele being detected is shown as heading to each panel with the expected band size. The genotype of the mouse is shown below each lane. HyperLadder II DNA Ladder (Bioline (Aust) Pty Ltd, Sydney, Australia) was used (M). Genomic DNA isolated from mouse tails PCR-amplified with specific primers. The PCR products were resolved on 1.5% AMRESCO agarose (Astral Scientific Pty Ltd, Sydney, Australia) and then visualized by SYBR Safe DNA Gel (Invitrogen, Carlsbad, CA, USA) staining. (DOCX) [file pone.0107931.s001.docx]

SUPPORTING INFORMATION

Data S1


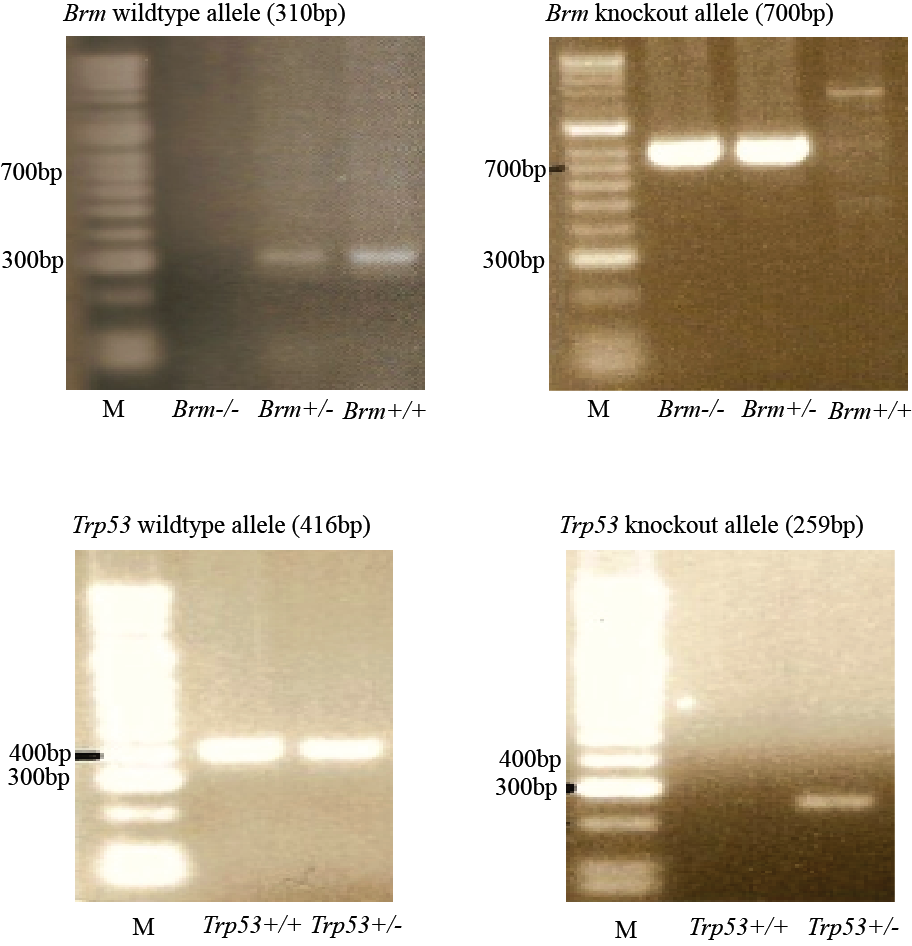


Figure S1. Genotyping of individual mice. Examples of PCR gels with the expected PCR products obtained during genotyping of individual mice. Each mouse was genotyped. The allele being detected is shown as heading to each panel with the expected band size. The genotype of the mouse is shown below each lane. HyperLadder™ II DNA Ladder (Bioline (Aust) Pty Ltd, Sydney, Australia) was used (M). Genomic DNA isolated from mouse tails PCR-amplified with specific primers. The PCR products were resolved on 1.5% AMRESCO agarose (Astral Scientific Pty Ltd, Sydney, Australia) and then visualized by SYBR® Safe DNA Gel (Invitrogen, Carlsbad, CA, USA) staining.

Methods S1

DNA was isolated as described (Herrmann, B.G. and Frischauf, A.M. 1987. Isolation of genomic DNA. Methods Enzymol. 152: 180-183). The mouse genomic DNA was PCR-amplified in 20µl of reaction mixture containing 1µl genomic DNA, 10µl Phusion master mix (Thermo Fisher Scientific, Scoresby, Victoria, Australia) and 10pmol of respective primers. To identify the *Brm* status, the first primer is the forward for both *Brm+/+* and *Brm-/-* PCRs: 5’-CTGAGTCATTTGCTATAGCCTGTG-3’, and the reverse primer for *Brm+/+*(310bp) is: 5’-CTGGACTGCCAGCTGCAGAG-3’ and the reverse primer for *Brm-/-* (700bp) is: 5’-CATCGCCTTCTATCGCCTTC-3’. To detect the *Trp53* genotype, the first primer is the forward for both *Trp53+/+* and *Trp53+/-* PCRs: 5’-CCCATGCAGGAGCTATTACACAT-3’ and the reverse primer for *Trp53+/+* (416bp) 5’-GATCTCTCGTGGGATCATTGTTT-3’ and the reverse primer for *Trp53+/-* (259bp*)* 5’-CCAGCCTAGACTGATGTTGACTTT-3. PCR was run on a T-100 Thermal Cycler (Bio-Rad, Sydney, Australia). PCR thermo cycling conditions were set in accordance with the protocol: 98°C for 5 min (1 cycle); 98°C for 30 sec, 55°C for 1 min, 72°C for 1 min (35 cycles); 72°C for 5 min (1 cycle).

Data S2

Histopathological changes to cornea

Four wild type mice were irradiated for 5 weeks, 6 for 10 weeks, 4 for 15 weeks and 6 for 20 weeks. By 5 weeks of irradiation some damage was observed with mild atrophy and mild parakeratosis in the central epithelium and mild hyperplasia with foci of parakeratosis and goblet cell metaplasia at the periphery. By 10 weeks atrophy was more marked with the central corneal epithelium being usually only 2 – 3 cells thick, compared to 5 cell layers in un-irradiated mice. Moderate parakeratosis was also present and a single case of epithelial down-growth. Hyperplasia of 6-7 cells thick continued to develop at the periphery. After 15 weeks the atrophy remained in the central region and basal cells had become damaged with vacuolation and parakeratosis being more advanced. Hyperplasia continued in the peripheral regions. The pathology was similar after 20, but more extreme by 25 weeks. All wild type mice had damage with 80% having atrophy and 33% ulcers. Metaplasia was present in 40% and hyperplasia in 20% of the mice. The hyperplasia in the peripheral zones was up to 10 cells thick, often containing minor down growth pegs with variable degrees of parakeratosis or cutaneous metaplasia (Table 1). In summary, the corneal epithelium of the eyes of wild type mice became atrophic, which failed to be repaired in the central region that received the highest exposure to UV. The peripheral more shielded regions however developed a hyperplastic response, presumably to provide new cells and repair the central region.

Data S3

Histopathological changes to stroma of the eye

The stroma was examined in the wild type mice exposed to increasing times of irradiation. The earliest detectable microscopic change was loss of stromal cells at 2 weeks (see manuscript). Damage was greater after 5 weeks of UV with the central stroma becoming oedematous and the loss of resident cells more prominent. At the peripheral zones, fibrovascular activity became observable at a level with the ciliary processes. In some cases fibrovascular twigs extended into the outer peripheral third of the corneal stroma. By 10 weeks of UV there was melanosis of the attached iris where cranial synechia occurred. The central stroma became necrotic and oedematous with clefts, voids and fibrovascular development that invaded into the necrotic stroma. In the peripheral regions fibrovascular twigs occupied the outer peripheral third and had commenced to invade into the central stroma. After 15 weeks of UV the central stroma contained collagenolytic clefts and voids infiltrated with a small number of neutrophils and macrophages. Only about half of the caudal stromal layer remained viable and exudate was attached to the Descemet’s membrane on the anterior chamber aspect. By 20 weeks of UV little viable stroma remained. The stroma was oedematous and necrotic with frequent collagenolytic clefts and voids in the matrix. Fibrovascular twigs had entered the central zone accompanied by increasing numbers of inflammatory cells. The peripheral region had increased active fibrovascular twigs and inflammatory cells. By 25 weeks of irradiation little viable stroma was present although fibrovascular twigs continued to form. In the peripheral regions the caudal half of the stroma was present and viable but did contain neo-angiogenesis and inflammatory cells accompanied by collagenous matrix formation. Identified stromal cells no longer displayed their original micromorphology. Some undoubtedly originated from the reparative fibrovascular tissue. Damage to the stroma appeared to be more extensive and to develop earlier than damage to the epithelium.
